# Clinical outcome and risk factors for subcutaneous emphysema in patients with lung cancer after video-assisted thorascopic surgery

**DOI:** 10.3389/fsurg.2022.956431

**Published:** 2022-09-02

**Authors:** Lei Wang, Yingxian Dong, Yanli Ji, Wenpeng Song, Chao Cheng, Mei Yang, Guowei Che

**Affiliations:** ^1^ Department of Thoracic Surgery, West China School of Nursing, Sichuan University, Chengdu, China; ^2^ Lung Cancer Center, West-China Hospital, Sichuan University, Chengdu, China; ^3^ Department of Thoracic Surgery, West China Hospital, Sichuan University, Chengdu, China

**Keywords:** clinical outcome, risk factor, subcutaneous emphysema, video-assisted thoracoscopic surgery, lung cancer

## Abstract

**Background and purpose:**

With the clinical application of minimally invasive surgery and concept of enhanced recovery after surgery, the incidence of postoperative complications in lung cancer patients has been significantly reduced. However, postoperative subcutaneous emphysema (SE) becomes the main factor affecting the early discharge of patients. The aim of this study was to analyze the clinical outcome and risk factors for postoperative SE in lung cancer patients.

**Methods:**

The clinical data of 414 lung cancer patients who were admitted to the Department of Thoracic Surgery, West China Hospital, Sichuan University from September 2021 to December 2021 were prospectively collected. The incidence, severity and treatment of patients who had SE, surgery approach, application of drainage tube and clinical information were analyzed.

**Results:**

The incidence rate of postoperative SE in patients with lung cancer was 33.09% (137/414) and mild cases accounted for the vast majority (30.19%, 125/414). Multivariate analysis indicated that male [odds ratio (OR) = 2.247, *P* = .014] and advanced age (OR = 1.021, *P* = .043) were main risk factors for postoperative SE in patients with lung cancer. Conservative treatment was the main treatment option for SE (98.5%, 135/137). The average hospital stay in the subcutaneous emphysema group (5.49 ± 4.41 days) was significantly longer than that in the non-subcutaneous emphysema group (4.44 ± 3.32 days) (*P* = .014) and no significant statistical difference in the average total hospital cost between the two groups (7,798.31 ± 1,414.85$ vs. 7,501.14 ± 1,605.18$, *P* = .072).

**Conclusion:**

Postoperative SE in patients with minimally invasive lung cancer is mainly mild, and conservative treatment is appropriate for most cases.

## Introduction

The progress of minimally invasive surgery and enhanced recovery after surgery (ERAS) has made the reduction of the incidence of postoperative pulmonary complications, especially pulmonary infection, and shortened length of stay (LOS) after surgery (1, 2). However, with the decrease of the incidence of postoperative serious complications, patients’ attention gradually shifted to postoperative comfort and returning to routine life as soon as possible. The symptoms after thoracic surgery are often the main factors that cause patients’ discomfort and anxiety. It has shown that the main postoperative symptoms after thoracic surgery are pain, cough, and fatigue (3, 4). Pain and cough management has been already studied and discussed, which means that patients with pain or cough will be less worried if these symptoms are handled properly. However, subcutaneous emphysema (SE) has gradually become one of the main postoperative complications after thoracic surgery, which was thought to be associated with surgery incision and air leakage, etc. Although it has little impact on clinical outcomes of patients, anxiety will be worse if SE exists and patients’ quality of life will be impacted (5, 6). At present, the incidence and risk factors of SE after minimally invasive surgery for lung cancer patients have not been reported, and only a few studies have been reported this (7), which is insufficient to clarify it. We prospectively analyzed the incidence, severity, risk factors, and treatment options of SE in lung cancer patients after video-assisted thorascopic surgery (VATS) to find out the causes of SE and provide clinical treatment strategies.

## Patients and methods

### Ethical review

Prior to submission, this study was licensed with the Chinese Clinical Trial Registry (ChiCTR2100046629). In addition, the participants signed a formal informed consent form. The research was presented using the STROCSS criterion.

### Inclusion and exclusion criteria

Data of 504 patients who underwent VATS for lung cancer in the Department of Thoracic Surgery, West China Hospital of Sichuan University from September 2021 to December 2021 were collected. Patients would be included if they (1) underwent VATS; (2) were diagnosed as primary lung cancer; and (3) had no history of severe chronic obstructive pulmonary disease (COPD), asthma, or cough before surgery. Patients would be excluded if they (1) underwent thoracotomy or pneumonectomy; (2) had reoperation after surgery due to bleeding or persistent air leakage; and (3) had a history of neoadjuvant chemoradiotherapy. Among the 504 patients, 55 patients’ pathological diagnosis were benign nodules, 35 patients’ clinical information were incomplete, and 414 patients were eventually included.

### Surgical approach

Single direction thoracoscopic lobectomy or segmentectomy (single-port or three-port) + systematic (or specific) lymph node dissection was applied (8). Underdeveloped fissures and intersegmental tissues were treated by mechanical staplers. Systematic lymphadenectomy was performed in all cases after the pulmonary resection (9). As for wound surface and bronchial stump, no special treatment was used.

### Chest tube management

In this study, single chest tube drainage was used in all patients. However, a total of 35 patients were inserted 18F silicon Foley catheter for chest drainage, which was through a port wound in the third or fourth intercostal middle axillary line and then descended toward the dorsal region (10). And 28F thoracic drainage tube was used in 379 patients, which was inserted at the seventh intercostal space. The same water-sealed chest drainage bottle was used in both groups after surgery, and negative pressure suction was not used (11). Twenty-four hours after surgery, chest tube removal was performed if the lung remained fully expanded from the chest x-ray and no air leak was observed in the water seal chamber. The chest tube could be removed safely even if the daily serous effusion was of a high volume (up to 450 ml/24 h) (12).

### Pain management

Patient-controlled analgesia (PCA) and thoracoscopic intercostal nerve blocks (TINBs) were used for postoperative analgesia (13, 14). Non-steroidal anti-inflammatory drugs (NSAIDs) were given to the patients if needed (15). In this study, 35 patients were treated with TINBs for analgesia and 379 patients were treated with PCA. All patients’ visual analogue scale (VAS) were less than 3 points.

### Endpoints for the study

The primary endpoint was the clinical outcome of SE. Since the severity of SE has not been discussed in any articles or guidelines, this severity classification is based on our experience in clinical work: Mild: emphysema is around the ipsilateral chest wall. Moderate: emphysema in ipsilateral and contralateral chest wall; Severe: emphysema in the chest wall and neck or face; Extremely severe: emphysema spread all over the body (abdomen and legs).

Risk factors of SE were analyzed as secondary endpoints. According to previous studies and clinical experience, age, height, sex, body mass index (BMI), percentage of the actual value of forced expiratory volume in one second and to the estimated value (FEV_1_%) and diffusing capacity of lung for carbon monoxide (DLCO), time of anesthesia, preoperative pulmonary disease, number of surgical incisions, drainage tube size, pleural adhesion degree, and surgical method were thought to be related to SE, which were collected for logistic regression (16).

### Statistical analysis

Statistical Package for the Social Sciences (SPSS) (Version 22.0, IBM, Armonk, NY, USA) was used to analyze the research data. The counting data were expressed by the actual number of cases and percentage. The Mean ± standard deviation (Mean ± SD) was used to represent the continuous variables that followed the normal distribution. The analysis method was the *t*-test of two independent samples. Median (minimum number, maximum number) was used to describe the variable data that did not obey normal distribution, and Mann–Whitney *U* test was used to analyze the comparison between groups of categorical variables. Chi-square test was used to analyze the comparison between groups of unordered categorization variables. The risk factors of SE were analyzed by univariate and multivariate logistic regression. *P* < .05 was taken as a marker to measure the significant difference between the two groups.

## Results

### Study population

A total of 414 patients were enrolled, with an average age of 53.83 ± 12.24 years; among 414 patients, 148 patients were male (35.7%, 148/414) and 266 were female (64.3%, 266/414). The average height was 161.40 ± 7.60 cm. The mean BMI was 23.08 ± 2.92. A total of 62 patients had a history of smoking (14.98%, 66/414); the mean FEV_1_% was 106.42 ± 16.57 and mean DLCO was 23.65 ± 3.59 ml/K pa/s. There were 129 patients (31.16%, 129/414) with cough symptoms before operation. Pulmonary disease occurs in 36 patients (8.7%, 36/414). A total of 187 patients (45.2%, 187/414) underwent single-port VATS and 227 patients (54.8%, 227/414) underwent three-port VATS; the average duration of anesthesia was 152.66 ± 55.15 min and mean operation period was 99.49 ± 44.56 min. The mean LOS was 4.79 ± 3.74 days. Average hospitalization cost was 7,599.48 ± 1,580.52 dollar. Thirty-five patients (8.5%, 35/414) were treated with 18F Foley catheter and 279 patients (91.5%, 379/414) were treated with 28F chest tube for chest drainage. All 414 patients were diagnosed as stage I adenocarcinoma according to the Union for International Cancer Control (UICC) (2018) lung Cancer staging standard. None of the patients had other postoperative complications requiring clinical treatment. According to the occurrence of SE, patients were divided into subcutaneous emphysema group (SEG) and non-subcutaneous emphysema group (NSEG), among which 137 cases were SEG and 277 cases were NSEG. Except for age (*P* = .003), gender (*P* = .000), and cough history (*P* = .036), there was no statistical difference in population characteristics between the two groups. Table 1 presents the characteristics of patients in SEG and NSEG.

**Table 1:** Population characteristics of patients in SEG and NSEG.

Index	NSEG (*n* = 277)	SEG (*n* = 137)	*P*-value
Age (year)	52.58 ± 12.26	56.35 ± 11.84	.003
Gender			
Male	81 (29.2)	67 (48.9)	.000
Female	196 (70.8)	70 (51.1)	
Smoking history			
Yes	36 (13.0)	26 (19.0)	0.109
No	241 (87.0)	111 (81.0)	
Comorbidities			
Yes	23 (8.3)	13 (9.5)	0.687
No	254 (91.7)	124 (90.5)	
Cough history			
Yes	77 (27.8)	52 (38.0)	.036
No	200 (72.2)	85 (62.0)	
Height (cm)	160.90 ± 7.75	162.42 ± 7.22	.056
BMI	23.15 ± 2.95	22.94 ± 2.87	0.483
Pulmonary function			
FEV_1_%	106.56 ± 16.90	106.12 ± 15.92	0.797
DLCO	23.64 ± 3.66	23.66 ± 3.45	0.954
Anesthesia period (min)	149.84 ± 52.58	158.34 ± 59.82	0.140
Operation period (min)	96.49 ± 43.17	105.54 ± 46.83	.052
Incision type			
One-port	118 (42.6)	69 (50.4)	0.135
Three-port	159 (57.4)	68 (49.6)	
Chest tube type			
18F	27 (9.7)	13 (9.5)	0.933
20F	250 (90.3)	124 (90.5)	
Pleural adhesion			
Mild	218 (78.7)	101 (73.7)	0.204
Moderate	51 (18.4)	27 (19.7)	
Severe	8 (2.9)	9 (6.6)	
Surgery approach			
Lob	81 (29.2)	49 (35.8)	0.173
Seg	83 (30.0)	38 (27.7)	
Wed	70 (25.3)	23 (16.8)	
Lob + Wed	17 (6.1)	14 (10.2)	
Seg + Wed	26 (9.4)	13 (9.5)	
LOS (day)	4.44 ± 3.32	5.49 ± 4.41	.014
Hospitalization cost (USD)	7,501.14 ± 1,605.18	7,798.31 ± 1,414.85	.072

SEG, subcutaneous emphysema group; NSEG, non-subcutaneous emphysema group; BMI, body mass index; FEV_1_, forced expiratory volume in one second; DLCO, diffusing capacity of lung for carbon monoxide; Lob, lobectomy; Seg, segmentectomy; Wed, wedge resection; LOS, length of stay; USD, USA dollar.

### Clinical characteristic and outcome of patients with SE

**Figure 1:**
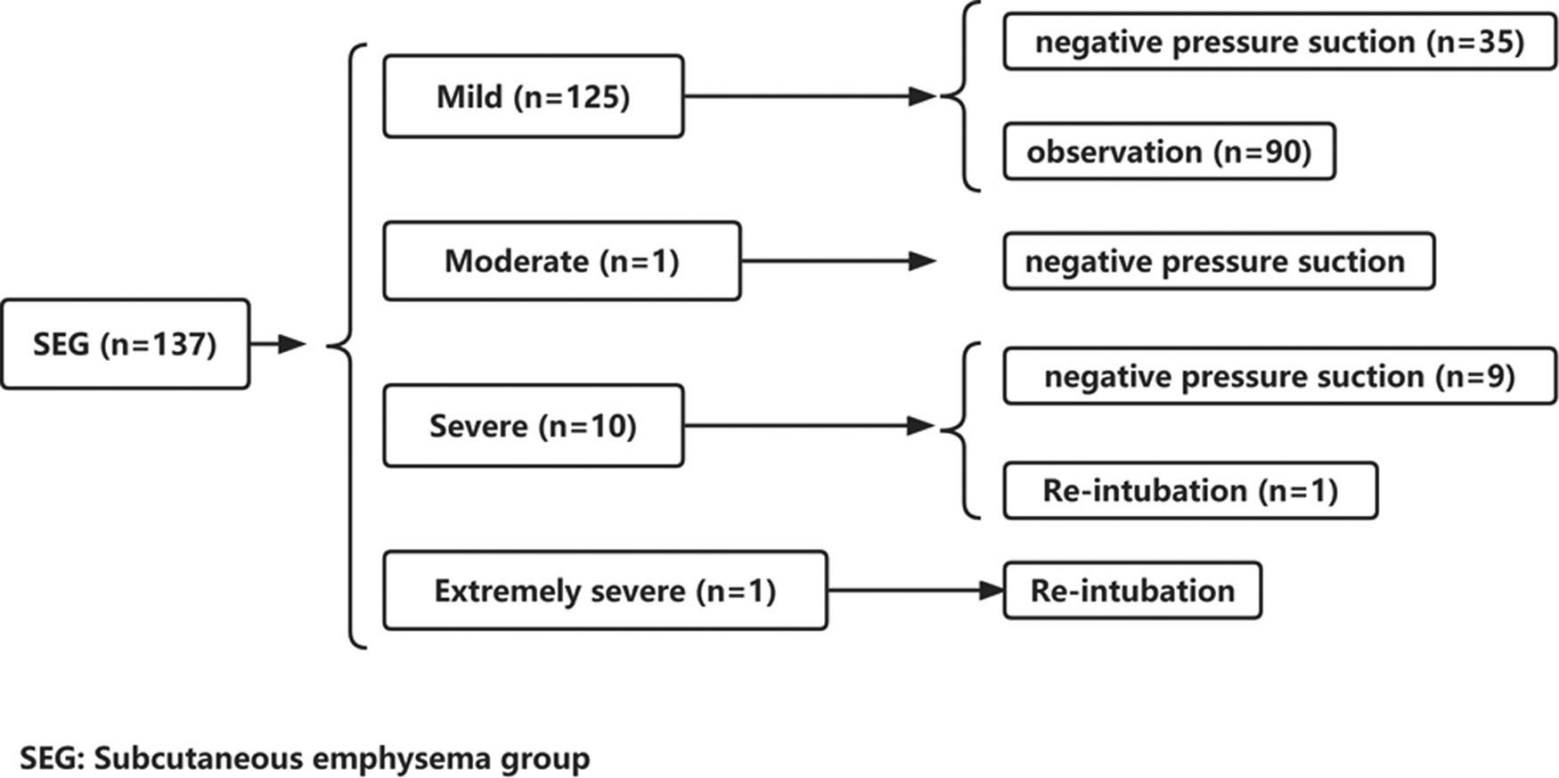
The proportion of different degree of subcutaneous emphysema and treatment method.

The incidence of postoperative SE in lung cancer patients was 33.09% (137/414). According to the severity, the proportion of mild patients was 91.24% (125/137); moderate patients 0.73% (1/137), severe patients 7.29% (10/137), and extremely severe patients 0.73% (1/414). Age in SEG was significantly higher than that in NSEG (56.35 ± 11.84 years vs. 52.58 ± 12.26 years, *P* = .003). The incidence of SE in male patients was statistically higher than that in female patients (45.72% vs. 26.31%, *P* = .000). The incidence of SE in patients with preoperative cough was higher than that in patients without cough (40.31% vs. 29.82%, *P* = .036). Average LOS in SEG was significantly higher than that in NSEG (5.49 ± 4.41 days vs. 4.44 ± 3.32 days, *P* = .014). There was no significant difference in hospitalization cost between the SEG and the NSEG (7,798.31 ± 1,414.85$ vs. 7,501.14 ± 1,605.18$, *P* = .072). Details can be seen in Table 1. Of 137 patients with SE, 90 patients (65.7%, 90/137) were treated with observation, 45 patients received negative pressure suction (32.8%, 45/137), and 2 patients underwent re-intubation (1.5%, 2/137), which can be seen in Figure 1.

### Multivariate binary logistic regression analysis of subcutaneous emphysema

**Figure 2:**
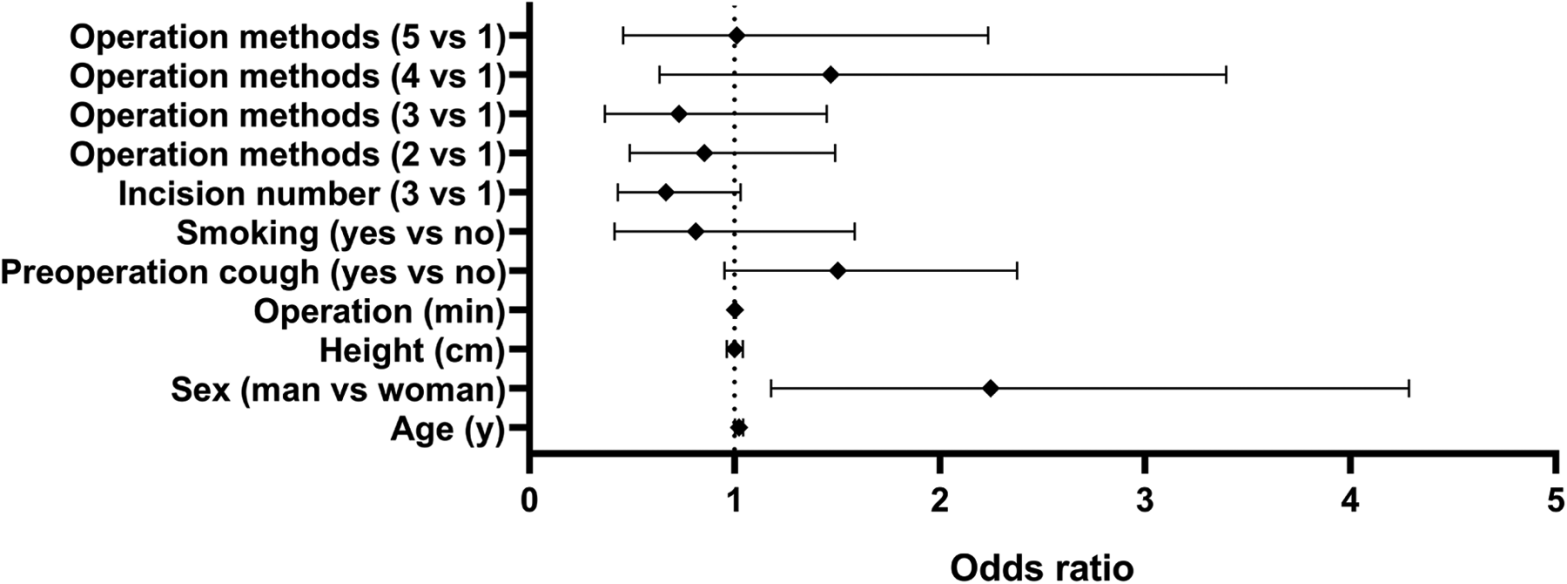
Odds ratio of operation methods, incision number, smoking history, cough, operation duration, height, sex and age for subcutaneous emphysema.

According to the results of the univariate analysis, elder age cough history and male may influence the incidence of SE (*P* < .05). These factors were included in the multivariate binary logistic regression analysis. The results show that the factors of elder age (*P* = .043) and male sex (*P* = .014) were independent risk factors of SE. Height, surgery period, preoperative cough, smoking history, incision type, and surgical approach were not risk factors for SE after VATS (see Table 2 and Figure 2).

**Table 2:** Risk factor analysis of subcutaneous emphysema.

	OR	*P*	95% CI
Age (year)	1.021	.043	1.001–1.041
Sex	2.247	.014	1.178–4.285
Height (cm)	1.000	0.982	0.9611.040
Surgery period (min)	1.001	0.595	0.996–1.007
Cough history	1.503	.081	0.950–2.377
Smoking history	0.811	0.539	0.415–1.585
Incision type	0.666	.067	0.431–1.029
Surgery approach			
Lobectomy	0.997	0.635	0.784–1.035
Segmentectomy	0.853	0.577	0.489–1.490
Wedge resection	0.729	0.366	0.367–1.448
Lobectomy and wedge resection	1.468	0.369	0.635–3.396
Segmentectomy and wedge resection	1.010	0.981	0.456–2.235

OR, odds ratio; CI, confidence interval.

## Discussion

In recent years, situation of lung cancer and thoracic surgery has gradually changed: first, the average age of patients has decreased and the detection rate of early-stage lung cancer has increased (17); second, thoracic surgery has been transformed into minimally invasive surgery (18), and the postoperative quality of life and survival rate of lung cancer patients have also been greatly improved (19). The integration of minimally invasive surgery and ERAS has greatly reduced perioperative complications of lung cancer patients, especially lung infection, and significantly shortened LOS (20, 21). These changes have led to the rising of patients’ requirements for surgery and postoperative recovery. Patients not only hope for a more comfortable perioperative period and rapid postoperative recovery, but also hope to improve their quality of life and return to work as soon as possible (22). However, patients with lung cancer may be anxious about the outcome of tumor treatment due to the aggravation of postoperative symptoms (23), including early postoperative pain, cough, pulmonary air leakage, SE, etc. The pain and cough are mainly caused by surgery and can be recovered by medication (13, 14, 24). While SE is easily misunderstood by patients as the tendency of poor operation or deterioration of their condition, which is more likely to cause anxiety of patients and their families, resulting in poor satisfaction and sometimes become adverse events. Therefore, to explore the occurrence and treatment of SE after VATS has become the focus of this study, which can not only improve our understanding of SE, but also help doctors and nurses to unify their views, to explain to patients and eliminate their concerns.

Previous studies and our clinical experience have shown that thoracic adhesion and undeveloped pulmonary fissure are risk factors for postoperative pulmonary air leakage and SE (25), and postoperative improvement of thoracic drainage tube, such as single drainage tube and small-size drainage tube, may lead to SE (10). However, our study found that the diameter of thoracic drainage tube and the adhesion and severity of pleural cavity were not the factors affecting the occurrence of SE. Even smoking history, pulmonary function (FEV_1_% and DLCO), anesthesia duration, presence of preoperative pulmonary disease, incision type, and surgery approach were not risk factors for SE. In this study, the incidence of postoperative SE in patients with preoperative cough was significantly higher than that in patients without cough, but multivariate analysis showed that cough [odds ratio (OR) = 1.503, *P* = .081] was not the risk factor of postoperative SE. The incidence of postoperative SE was only correlated with elder age (OR = 1.021, *P* = .043) and male gender (OR = 2.247, *P* = .014). Male (OR = 2.247, 95% CI, 1.178–4.285) and elderly (OR = 1.021, 95% CI, 1.001–1.041) patients with lung cancer were more likely to have SE after surgery.

This study showed that 33.09% of patients had SE after VATS, 91.24% of patients had mild emphysema, 7.29% of patients had severe emphysema, and each moderate and extremely severe emphysema occurred in one patient (0.73%). Among the 137 patients with SE, only two patients needed re-intubation, 45 patients needed negative pressure suction therapy, and 90 patients only needed observation to recover. These results indicated that postoperative SE is common, mostly mild, and observation and negative pressure suction are the main means of treatment which means that there is no need for further worry about SE. The occurrence of postoperative SE was the main factor leading to the prolongation of the LOS, but the average hospitalization cost showed no statistical difference between the SEG and the NSEG.

Theoretically, pleural adhesion is the main risk factor leading to postoperative lung leakage and SE, but this result was not found in this study. Analysis of data showed that among 414 patients with lung cancer, 17 patients (4.11%) had severe thoracic adhesion, only 78 patients (18.84%) had moderate adhesion, and 319 patients (77.05%) had mild adhesion. SE occurred in 9 of the 17 patients with severe pleural adhesion (pleural atresia) and in 8 of the 17 without. Neither of them can indicate whether pleural adhesion or its severity affect the occurrence of postoperative SE. These results need to be supported by larger and multi-center clinical studies.

In our study, male and elder age were thought to be risk factors of SE, so we need to pay more attention to these people in case of their worsen anxiety. Male and elder age may be associated with poor pulmonary function that has been reported before (26). However, in this study, we did not find that poor FEV_1_% was statistically significant, which may be caused by insufficient sample size. In some studies, negative suction was used for prevention of air leakage and SE (27). Whether early negative pressure suction for chest drainage after surgery can reduce the occurrence or severity of SE requires further clinical research.

There are some limitations in this study. First, other factors that might affect SE, such as use of negative pressure suction, postoperative cough frequency, and daily activities, were not analyzed in this study as this information was not available in the medical records of all patients. Second, this study was not a randomized controlled trial; therefore, it might be affected by other factors. Third, the sample size of this study was relatively small. Multi-center studies with a large sample size are needed to validate the results of this study.

## Conclusion

The incidence of SE after VATS is high, but patients with SE are mainly mild to moderate, and the treatment is conservative observation. Male and elderly patients are considered risk factors for SE. Multi-center studies with a large sample size are needed to validate the results of this study.

## Data Availability

The original contributions presented in the study are included in the article/Supplementary Material, further inquiries can be directed to the corresponding author/s.
